# SES-of-Origin and BMI in Youth: Comparing Germany and Minnesota

**DOI:** 10.1007/s10519-018-9938-7

**Published:** 2018-11-29

**Authors:** Wendy Johnson, Elisabeth Hahn, Juliana Gottschling, Franziska Lenau, Frank M. Spinath, Matt McGue

**Affiliations:** 1 Department of Psychology, University of Edinburgh, 7 George Square, Edinburgh, EH8 9JZ UK; 2 Department of Psychology, Saarland University, Saarbrücken, Germany; 3 Cognitive Science & Assessment, University of Luxembourg, Luxembourg City, Luxembourg; 4 Jugendwerk St. Josef – Haus Maria Rosenberg, Waldfischbach-Burgalben, Germany; 5 Department of Psychology, University of Minnesota, Minneapolis, USA; 6 Department of Epidemiology, Biostatistics and Biodemography, University of Southern, Denmark, Odense, Denmark

**Keywords:** SES-of-origin, BMI, Obesity, Overweight, Youth, National differences

## Abstract

Increasing obesity is a world-wide health concern. Its most commonly used indicator, body mass index (BMI), consistently shows considerable genetic and shared environmental variance throughout life, the latter particularly in youth. Several adult studies have observed less total and genetically influenced variance with higher attained SES. These studies offer clues about sources of the ‘obesity epidemic’ but analogous youth studies of SES-of-origin are needed. Genetic and environmental influences and moderating effects of SES may vary in countries with different health policies, lifestyles, and degrees/sources of social inequality, offering further clues to the sources of the obesity epidemic. We examined SES-of-origin moderation of BMI variance in the German TwinLife study’s cohorts assessed around ages 5, 11, 17, and 23–24, and in the Minnesota Twin Family Study’s (MTFS) 11- and 17-year-old birth cohorts assessed longitudinally around ages 11, 17, and 23–24, comparing male and female twins and their parents. Age for age, both sexes’ means and variances were greater in MTFS than in TwinLife. We observed that SES generally moderated genetic influences, more strongly in females, similar to most adult studies of attained-SES moderation of BMI. We interpreted differences in our SES-of-origin observations in light of inevitably-missing covariance between SES-of-origin and BMI in the models, mother-father and parent–offspring BMI correlations, and parental attained-SES–BMI correlations. We suggest that one source of the present obesity epidemic is social change that amplifies expression of genes both constraining SES attainment and facilitating weight gain.

**Electronic supplementary material:**

The online version of this article (10.1007/s10519-018-9938-7) contains supplementary material, which is available to authorized users.

Overweight and obesity are increasing worldwide health concerns. The World Health Organization (WHO; WHO 2017) estimated that, in 2016, 39% of adults (39% of men, 40% of women) over 18 years old were overweight, and one-third (11% of all men and 15% of all women) of those were obese. The WHO reported that the 2016 rates were nearly triple those in 1975 and showed no signs of slowing. The terms ‘overweight’ and ‘obesity’ refer to accumulation of abnormal or excessive adipose tissue that may impair health, with obesity being more extreme and its associated risks thus greater. Obesity is associated with cardiovascular diseases such as heart disease and stroke (the leading cause of death in 2015 and 2012 according to the WHO), Type 2 diabetes (also experiencing sky-rocketing rates worldwide), musculoskeletal diseases such as osteoarthritis, and many of the most common cancers, including breast and prostate.

Importantly, obesity is usually preventable [Global Burden of Obesity Collaborators (GBOC) 2017; WHO 2017], suggesting that risks and prevalences of the diseases associated with it can be effectively limited by preventing obesity in the first place. Development of overweight inevitably precedes obesity because excess adipose tissue accumulates over time. Consistent with this, rates of overweight and obesity in children are lower than in adults, and lower in adults of younger than older ages, at least until late adulthood (GBOC 2017). Nonetheless, obesity rates in children have also been increasing rapidly (WHO 2017). GBOC (2017) estimated that, in 2015, 5% of children under 18 were obese, and this rate had increased in recent years more rapidly than that in adults. Though data on age-related patterns of obesity development are sparse, the available evidence suggests that, age for age, more recently born cohorts are accumulating adipose tissue more rapidly, The earlier in life adiposity becomes excess, the more likely a person is to become obese and to encounter its associated health problems (e.g., Emmett and Jones 2015; Johnson et al. 2015; Langstrom et al. 2008). This implies that it is especially important to identify and understand the processes underlying adiposity accumulation in childhood and youth.

There is very consistent evidence for substantial but far from deterministic genetic influences on propensities both to carry and accumulate adipose tissue throughout the lifespan (e.g., Albuquerque et al. 2017; Fernandez et al. 2012; Hjelmborg et al. 2008; Sillventoinen et al. 2010). This is also the case for factors that can influence adiposity accumulation such as food preferences (e.g., Reed et al. 2006), metabolic rates (e.g., Muller and Geisler 2017), and physical activity levels (e.g., de Geus et al. 2014). Both total amounts and proportions of variance attributable to genetic influence, however, vary considerably with age, over time, with country level economic development and rate thereof, and ambient obesity level (e.g., Min et al. 2013). Along with the current ‘obesity epidemic’, the consistent evidence for substantial non-shared environmental as well as genetic influences, and for shared environmental influences in children and youth, this attests to the importance of understanding the involved genetic and environmental interplay (e.g., Albuquerque et al. 2017).

## Obesity prevalence patterns, economic wealth and socioeconomic status, and Simpson’s Paradox

Though obesity is a problem worldwide, rates vary considerably among countries, and often among regions within countries. Countries and regions within them with higher levels of education and greater economic wealth tend to have higher rates (GBOC 2017; WHO 2017), though there are also many exceptions to this (for example, Samoa runs a very high rate, while Japan’s rate is low). Perhaps seemingly paradoxically, however, within most ‘economically developed’ regions with high rates, individuals with greater education and personal wealth [higher socioeconomic status (SES)] tend to have lower rates of obesity. Such situations, in which group–level correlations disappear or have the opposite direction among individuals in the groups, are generally counter-intuitive, but they occur with some frequency in socially and medically important variables. In the field of statistics, they are labelled examples of ‘Simpson’s Paradox’ (Blyth 1972), in recognition of their first technical exposition (Simpson 1951).

These situations apparently seem counter-intuitive because, no matter how many times the mantra that correlation does not imply causation is repeated, even well-trained researchers tend to reach first for directly causal explanations for observed relations between variables. They often assume that, as would be the case if one variable directly caused the other, what is observed for individuals within groups, should also be true at the group level. All too often, however, some unmeasured third variable has a causal influence on both variables. In such situations, observed associations are often termed ‘spurious’. This does not mean that they are not valid, but rather that they are not informative about cause. The problem then becomes to identify the actually causal variable(s). Simpson’s Paradox occurs when one or more of these actually-causal variables acts differently on individuals than on relevant grouping variables. Units of time measurement often function in this manner. Common examples involve success rates. One person’s can be consistently better than another’s each year, yet lower over a 5-year period. This happens when the numbers of opportunities for success vary from year to year, and, as well, the consistently less successful person happens to be relatively more successful in years when there are many opportunities and the other happens to have things go the other way. Donald Trump’s election to the US Presidency despite losing the popular vote is another example of Simpson’s Paradox: aggregated at the state level according only approximately reflecting the numbers of individual voters in each state, the count was rather different than when tallied at the individual level.

More relevant to obesity development, genetic and environmental influences can confound observed associations to create Simpson’s paradoxes when they cluster in families (or more broadly in society due to gene–environment correlations) differently than in individuals. For example, Armour and Haynie (2007) observed that adolescents experiencing earlier ‘sexual debut’ (first sexual intercourse) were more likely to behave delinquently over the next few years. They inferred that getting adolescents to defer sexual initiation would help keep them out of trouble. Using the same sample, however, Harden et al. (2008) observed that, controlling genetic and shared environmental influences by examining the same association within twin pairs, twins who had sexual intercourse earlier were *less* likely to behave delinquently over the same period. As they explained, Armour and Haynie’s observed association consisted of any actual effect of age of first intercourse on delinquent behavior as well as all unmeasured variables that differed among families and also affected both timing of first intercourse and delinquent behavior. Something in genetic or shared environmental background that affected both timing of first intercourse and tendency to behave delinquently differently in different social groups or families of adolescents reversed the observable direction of association.

A textbook example of Simpson’s Paradox suggests strongly that there may be similar kinds of genetically and environmentally confounded explanations for the contrasting associations between socioeconomic indicators and obesity rates and trends at national/regional and individual levels. Meat consumption is associated with longer life expectancy at the national level, but shorter life expectancy at the individual level (e.g., Skrondal and Rabe-Hesketh 2004). This appears to be because, among wealthier nations, both health care and meat are more available and affordable than they are in less wealthy nations, but, at the individual level within countries, large amounts of meat consumption are not healthy. All kinds of food products tend to be more available in wealthier countries, and all too often the least healthy are cheapest and the healthiest foods are difficult to find in relatively deprived areas. This suggests that studying the relations between genetic and environmental inter-relations between obesity-related measures and socioeconomic status during childhood, adolescence, and adulthood within and between nations may be helpful in understanding the emergence and trends in the ‘obesity epidemic’.

Several twin studies have observed generally lower mean levels of and less total and genetic variance in measures related to obesity among adults of higher SES within countries. It is much easier to find evidence of such moderating effects than it is to illuminate how they arise, yet doing the latter is ultimately more important to understanding any phenomenon in which they are involved. Working with United States data and income as the measure of SES, Johnson and Krueger (2005a) observed that these observations held even after controlling health insurance coverage. In a follow-up paper, they observed that perceived control over life was associated very similarly with both mean relative level of BMI and its total and genetic variance (Johnson and Krueger 2005b). They interpreted this as suggesting that both low SES and circumstances that limit ability to maintain a feeling of control over life act as stressors that affect both food- and exercise-related behaviors and metabolic processes related to fat accumulation, thus contributing to obesity development. Such a pattern would be consistent with the robustly observed associations between low SES and high stress levels (e.g., Baum et al. 1999). Given the genetic correlations Johnson and Krueger also observed between perceived control and relative obesity, they went on to suggest that the same genes that tend to facilitate ability to find environmental niches allowing a sense of control over life also facilitate exerting that control to maintain healthy body weight, and that experience with failing to find such niches may contribute to a sense of futility.

Working with Danish data and education as the measure of SES, Johnson et al. (2011) observed similar patterns of lower mean levels and total variances of BMI with higher SES. There was greater power in this sample than in previous studies, so observations were more nuanced and sex differences were addressed. In females, shared and non-shared environmental as well as genetic variances were lower among those with higher levels of education, but, in males, only the two environmental components were moderated this way—genetic variance was not moderated. In both sexes, genetic correlations were minimal and shared environmental variance was particularly variant with education level. This suggested nationally-related differences between the United States and Denmark or measure-related differences between income and education as indicators of SES, or both. Education consistently shows shared environmental influences throughout the lifespan, unlike income and many other variables (e.g., Freese and Jao 2017), which may have been one reason for the difference, but lower income disparities and much more uniform access to medical care in Denmark than the United States may also have been involved.

Returning to a United States setting and examining education, household income, and neighborhood-level socioeconomic advantage separately, Dinescu et al. (2016) observed the same general patterns of less genetic variance and stable but minimal gene-environment correlation with higher SES as did Johnson and Krueger (2005a) for each of their SES measures. They also observed less non-shared environmental variance with higher SES, especially for neighborhood-level socioeconomic advantage. Their sample was larger than in the previous study, so greater power may have been involved. Like Johnson and Krueger (2005b), they interpreted these patterns as indicating that something about low SES brings out expression of genetically-related vulnerabilities to weight gain. To address the lower non-shared environmental variance, they noted their measure of neighborhood-level socioeconomic advantage. Unlike education and income, which are personal resources that can be used to select and shape environments, neighborhood-level socioeconomic advantage is a community environment that affords and constrains opportunities to make use of personal resources. The similarity in overall observed pattern coupled with additional moderation of non-shared environmental influence suggested intertwined genetic and environmental influences, and stronger impact of neighborhood-level socioeconomic advantage than education and income on non-shared environmental variance. This is consistent with the idea that, though people can and do select and shape their environments, once selected, those environments may have more power (very literally!) to shape them.

Two studies have also made use of polygenic risk scores to assess SES moderation of BMI in American adults. Liu and Guo (2015) observed increasingly strong associations between polygenic risk scores and BMI with lower ‘cumulative advantage’ (SES tracked across the lifespan using mid/late adult-reported father’s occupation to represent childhood, attained education to represent young adulthood, and wealth to represent current later-life status), and that, at the highest level of cumulative advantage, the association did not reach significance. They did not test directly for moderation, but the trend they observed was consistent with that observed in all the twin studies. Coleman et al. (2018) examined associations between (quite different) polygenic risk scores and both BMI and change in BMI in UK adolescents from ages 11–16 years, as well as sex-specific, social environment, SES, and parental style effects. Polygenic risk scores were associated with higher BMI at both ages, and with significantly increase from ages 11–16 in girls (but not boys), but their interaction was not significant. Lower SES was associated with higher BMI at age 11, but SES had no significant moderating effects. In both these studies, however, polygenic risk scores accounted for small proportions of variance (~ 5%), making it difficult to assess their consistency with each other and with the quantitative genetic twin study results.

These studies of SES and obesity-related measures suggest considerable consistency in patterns that may underlie and help explain the ‘obesity epidemic’, though more are clearly needed. As well, they suggest presence of the kinds of genetic and environmental confounds for which Harden et al. (2008) found evidence in sexual initiation. That is, the psychological importance of SES may largely be its social status rather than the amount of objective economic resources (e.g., Campbell et al. 1976; Michalos 1985). Low social status tends to be associated with relatively little control over life and greater stress, both of which leave people vulnerable to poor choices about health behaviors, including diet and exercise. When this is compounded by reduced availability and greater cost of healthy food choices in areas where low-SES people tend to live, greater expression of genetic vulnerabilities to obesity in those people seems plausible. Lack of economic resources, per se, would not be the cause, however (e.g., Diener and Oishi 2000), and such patterns may vary in informative ways from country to country. Prior studies were also all conducted in adults, and we are aware of no such studies to date in children or adolescents. Given that obesity in individuals accumulates over long periods of time and rates are increasing in children throughout the world, developmental studies of the moderating effects of SES on measures related to obesity will likely prove valuable as well, and should be carried out in a variety of countries as well as replicated within countries.

In adults, SES reflects the accumulation of the personal and economic resources generated by the opportunities a person has encountered and utilized to find a ‘place in the world’ that offers a means of financial support and a platform on which to make use of skills and abilities (Bradley and Corwyn 2002). Opportunities encountered are always at least somewhat dependent on the resources available during childhood, but utilization is independent, to varying degrees, especially after childhood. Children, however, grow up in the environments of, with the resources conferred by, their parents’ adult attained SES’s. Given robust evidence of genetic influences on adult attained SES and its equally robust observed (phenotypic) associations with many environmental contexts, personal characteristics, and life outcomes, SES may be a particularly strong source of trans-generational gene-environment correlation: in most families, genetically influenced parental characteristics such as intelligence, achievement motivation, assertiveness, self-control, etc., act to select and shape a ‘package’ of environmental features (local school quality, air and water quality, crime levels, access to ‘green space’, medical care, nutritious foods, etc.), and parents have passed both the genes contributing to the characteristics that helped them accumulate this package and the package itself on to their kids. It is hard to imagine environmental reinforcement of those genetically influenced personal characteristics not being common. Nonetheless, during childhood, SES-of-origin is an environment that has been ‘assigned to’ people to much greater degree than is SES attained in adulthood.

This creates an important methodological limitation for studies exploring SES-of-origin moderation of genetic and environmental variance components of personal characteristics that is seldom noted in such studies. Members of twin pairs growing up together and with their biological parents experience the same SES-of-origin and also inherit their parents’ genes to basically the same degrees (half the genes on which humans tend to vary, with MZ twins inheriting effectively the same half and DZ twins inheriting about 50%-overlapping halves when parents do not assort on the relevant trait). Thus any direct main effects of SES-of-origin on the personal characteristic cannot be distinguished from covariance between them. Even when parents invest differently in one child than another, this is generally to reinforce or remediate some genetically influenced child characteristic, thus inherently creating negative gene-environment correlation. The combined impact of any direct main effects of SES-of-origin and the gene-environment covariance is all modelled as direct main effect, as if it affected everyone uniformly. Actually, however, the covariance can have far from uniform effects on everyone. For example, it can include population stratification due to differences in gene frequencies that contribute to socially relevant performance capacities (e.g., intelligence), overt social discrimination on arbitrary personal characteristics not related to actual performance capacities (e.g., skin color). It can also or instead include specific environmental circumstances such as community resources and risks that suppress or enhance genetic expression of vulnerabilities or advantages to different degrees at different levels of exposure. Any of these may be openly and optionally available to all, imposed arbitrarily on some, or anything in between, which may impact the relations between SES-of origin and the twins’ levels of the personal characteristic, too (Johnson 2007). The result is that the part of the variance in that personal characteristic that is not modelled is precisely the part that is most relevant to the observed association between SES and outcome characteristic that aroused interest to begin with (Purcell 2002). At present, it is possible only to address this very indirectly, outside this and other models, by examining mean levels of the trait of interest at various levels of the moderator and by quantifying variance not modelled.

## Measuring overweight and obesity

Understanding the emergence of the ‘obesity epidemic’ motivated the study reported here. But, as noted above, obesity emerges in individuals gradually through accumulating overweight over some period of time that unfortunately increasingly includes even young childhood for many. Thus, though doctors and epidemiologists define cut-offs on measures of body adiposity to indicate overweight and obesity levels to be addressed clinically, the underlying adiposity dimension is continuous and these cut-offs arbitrary. Many ways of assessing extent of body adiposity have been developed, but most of them require expensive equipment and considerable assessment time. This limits availability and encourages reliance on simpler measures, which, unfortunately, tend to be less accurate. Body Mass Index (BMI; kg. weight/m^2^ height) is particularly readily accessible and is in most common usage. The WHO has established thresholds of 25 and over to define overweight and 30 and over to define obesity in adults. At the general-population statistical level at least in western countries, these BMI cut-offs track quite well with more precisely measured overweight and obesity rates (though lower cut-offs appear to be appropriate for Asian populations). At the individual level, however, they are far less accurate. Their use for this purpose has received considerable criticism, but they remain standard.

Establishing definitions of overweight and obesity in children and adolescents is considerably more difficult because typical healthy BMI at birth is about 13, and children grow at very different rates. Clinicians have long maintained charts of normative distributions of height and weight by age and sex for use in routine children’s physical exams, and both the US Center for Disease Control (CDC) and the WHO have used extremes of these distributions to develop recommended cut-offs for definition of children’s overweight and obesity.

The CDC recommends defining overweight as the 85th percentiles and obesity as the 95th percentiles of distributions in the 1977 National Center for Health Statistics growth charts for US children and adolescents. These were intended to represent the full US population at the time (Kuczmarski et al. 2000), and can arguably be considered to precede the ‘obesity epidemic’. The WHO commissioned the Multi-Centre Growth Reference Study to compile growth charts (Borghi et al. 2005) based on 8500 children following recommended health practices in a wide variety of ethnic and cultural contexts, initially collecting data from 1997 to 2003, but updating these with new data in 2007. The adult BMI cut-offs of 25 to define overweight and 30 to define obesity roughly corresponded to one and two standard deviations above the mean, respectively, at age 19, so the WHO applied these cut-offs to their 2007 children’s data for all ages, a time after the obesity epidemic had become established. Indicated prevalences of overweight and obesity using these two definitions tend to be similar but not identical.

## This study

Two twin studies of youth and their parents are well-positioned to offer comparison of German and American obesity and overweight prevalences under the CDC and WHO guidelines. Such information is largely descriptive, but it serves important purposes in all behavior genetic analyses, especially ones such as this comparing samples in different environments. These two studies are also well-positioned to compare moderating associations of SES on genetic and environmental influences on the full distributions of BMI in the two countries, as well as to examine its inter-generational transmission and developmental patterns from ages 5 to 24, because they have very similar ages of assessment and measures. The latter are rather rare, yet particularly important in illuminating factors contributing to gene-environment interplay—a crucial step beyond noting evidence of its presence. The purpose of this study was to take advantage of these data to explore how they might illuminate the developmental processes underlying the present obesity epidemic. In particular, we addressed the following questions.

1. How do BMI phenotypic mean and variance levels, rates of overweight and obesity compare in the two sexes and two countries in youth of various ages and their parents?
2. To what extent do these rates differ in children based on WHO and CDC data?
3. How similar is BMI in parents and offspring of various ages, and how does this similarity compare in the two sexes and two countries?
4. How do proportions of genetic and environmental variance vary with age in youth and compare in the two sexes and two countries?
5. How does SES relate to BMI in the two sexes and two countries in youth of various ages and their parents?
6. To what degree does SES of-origin moderate genetic and environmental influences on BMI in youth of various ages in the two sexes and two countries, and how do any patterns compare with those in adults?
7. Can these patterns help explain the increasing world-wide prevalence of obesity and some of the cross-national differences?

## Method

### Samples

Our German data were the first assessment of TwinLife, a prospective cohort-sequential longitudinal study of same-sex twins and their families (Diewald et al. 2016; Hahn et al. 2016; Lang and Kottwitz 2017; Lenau and Hahn 2017). The study includes four twin birth cohorts, each consisting of about 1000 pairs of approximately equal numbers of monozygotic (MZ) and dizygotic (DZ) twins, their parents, and one additional sibling, if available. Sample size was targeted by balancing the practicalities of recruiting and surveying against need for analytical power. The cohorts were recruited using stratified random sampling based on communal registrations in communities of varied size spread randomly throughout Germany, from birth years 2009–2010 (C1), 2003–2004 (C2), 1997–1998 (C3), and 1990–1993 (C4). Across the four cohorts, 55.9% of those deemed eligible participated, with participation somewhat higher in the younger cohorts than the older. Overall, sample demographics were highly representative of the German population, except that participating parents were somewhat better educated than average in the population (Lang and Kottwitz 2017). All cohorts were assessed in 2014–2015, making C1 about 5 years old, C2 about 11, C3 about 17, and C4 about 23. Yearly follow-up assessments are planned for a total period of 6 years, so that the first three cohorts’ follow-up ages align with the initial assessment ages of the adjacent cohorts, but none of these data are yet available. In total, study-relevant data were available for 4092 twin pairs, 3887 (95.0% of families) biological mothers, and 2545 (62.2% of families) biological fathers, though maternal participation ranged from 98.7% in C1 to 93.2% in C3 and C4, and paternal participation declined more sharply from 69.8% in C1 to 49.9% in C4. Overall, 54.8% of the twin pairs were female, with more equal numbers in the younger cohorts, and 45.8% were MZ, with lower proportions in the younger than the older cohorts (consistent with greater use of in vitro fertilization in more recent years). Table 1 shows sample detail by cohort, for both TwinLife and our American data. Because the latter did not include siblings of the twins, we did not make use of TwinLife’s sibling data in this study. Families were assessed in person at home by professional survey interviewers, and also completed written surveys. The interview assessment took about 3 h per family, and included a wide variety of topics and measures not used in this study (Hahn et al. 2016).

**Table 1:** Sample characteristics

TwinLife				
Cohort	N	Female (%)	MZ (%)	Pairs missing zygosity
1—Age ~ 5 years				
Participating mothers	995			
Participating fathers	704			
Twin pairs	1008	51.6%	43.1%	3
2—Age ~ 11 years				
Participating mothers	987			
Participating fathers	711			
Twin pairs	1041	52.1	40.4	2
3—Age ~ 17 years				
Participating mothers	987			
Participating fathers	639			
Twin pairs	1059	57.3	47.0	1
4—Age 23–24 years				
Participating mothers	918			
Participating fathers	491			
Twin pairs	984	58.1	53.3	1
MTFS				
11-YO cohort				
Participating mothers	1245			
Participating fathers	1235			
Twin pairs, age 11	1260	51.0	62.5	0
Twin pairs, age 17	779	51.4	63.3	0
Twin pairs, age 24	536	52.1	62.9	0
17-YO cohort				
Participating mothers	626			
Participating fathers	622			
Twin pairs, age 17	626	53.8	65.7	0
Twin pairs, age 24	183	0	62.2	0

*MZ* monogygotic. Cohorts based on twin-pair year of birth in TwinLife, twin-pair recruitment age in MTFS. Ages for MTFS refer to follow-up assessment age. Height and weight were not assessed for females at age 24 in the 17-YO cohort

The Minnesota Twin Family Study (MTFS; Iacono et al. 1999; Iacono and McGue 2002) provided the American data. As this sample was recruited based on Minnesota birth records, required participants to live within a day’s drive of the University of Minnesota—Twin Cities, and obesity rates in the US vary quite a bit by state, from here on we refer to these as Minnesota rather than American data. Like TwinLife, MTFS is a longitudinal cohort-sequential study. It consists of two age-based same-sex twin cohorts and their parents, one assessed initially at age 11 years, and the other at age 17. They were recruited after locating over 90% of the twin births in Minnesota in the years 1972–1979 for the older cohort and 1981–1984 and 1988–1994 for the younger cohort. Since initial assessment, the older cohort has been reassessed at target ages 20, 23–24, 27, and 29 years, and the younger at target ages 14, 17, 20, 23–24, 27, and 29. More than 80% of recruited families participated initially, and follow-up participation rates have been over 90% (McGue et al. 2014). For this study, we used data from the ages 11, 17, and 23–24 assessments to match the ages assessed in TwinLife. MTFS families were generally representative of the Minnesota population in the years of the twins’ births. Mothers averaged about 13.5 years of education, fathers about a year more. These averages were slightly higher in the younger than the older cohort, consistent with generally increasing educational attainment over time in the state. Average household occupational level in the families was slightly over the skilled ‘blue collar’ level, again somewhat higher in the younger than older cohort, consistent with state data on such changes. It spanned the range, however, from highly professional to semi-skilled and unemployed. Over 80% of families that declined to participate did complete a brief mail or telephone survey. This indicated that participating parents were slightly (on average, 0.3 years) more educated than those not participating, but the families did not differ significantly in self-reported mental health.

In total, 1886 families participated (626 in the older, 1260 in the younger cohort), including 1199 MZ twin pairs and 687 DZ pairs, of which 51.9% were female (like TwinLife, slightly more equally distributed in the younger cohort). Relevant data were available for 98.0% of biological mothers, and 82.6% of biological fathers (again, like TwinLife, with slightly higher availability rates of both in the younger than older cohort). Families were assessed on a wide variety of characteristics during a day-long assessment in the study’s lab at the University of Minnesota. Mothers also completed a telephone interview about the family, and all participants completed mail-in questionnaires. This assessment as well included a wide variety of measures not used in this study.

Our demographic research questions comparing basic rates of obesity and BMI–SES correlations in children of various ages, the two sexes, and their parents in the two countries could more effectively be addressed in census-based surveys. Addressing them in this study was important, however, for two reasons. First, the degree to which our results were consistent with observations from broader samples attested to the representativeness of our samples, and thus the likely validity of our behaviour genetic analyses, Second, other sources of data allowing explicit intergenerational comparisons of obesity rates are rare, and most other sources do not compile data on variance but only compare mean levels and proportions falling above cut-offs. Yet such data afford a valuable perspective on how obesity rates are accumulating over time, and thus how it may spread throughout a population.

### Measures

Height and weight data were compiled in both samples by study interviewers, via self-report in TwinLife, usually direct measurement in MTFS. We used these to calculate BMI, adjusting TwinLife twin BMI data for age and age^2^ separately in each sex and cohort, and MTFS twin BMI data controlling birth year as well as age within each assessment age. In both samples, we adjusted parental BMI separately in each sex across their full age ranges (adding twin birth year for MTFS because it varied within age cohort), and capped outliers in all groups. Because some of the group distributions were positively skewed to a degree that could affect analytical robustness (notably, standardized skewness coefficients ranged from 1.0 to 1.80), we analysed both ‘raw’ age-adjusted and log-transformed data. Effects were consistently slightly smaller in the transformed data but always in the same direction, indicating high robustness in the untransformed data. We thus report untransformed results, to keep interpretation as straightforward as possible.

Both samples had measures of parental occupation, educational attainment, and income on which to base composite measures of family SES (SES-of-origin for the twins). TwinLife had two measures of educational attainment for each parent. One measured secondary educational level in the German school system in detail. The other was the 1997 International Standard Classification of Education code based on the German data. After-tax household income was assessed using the current Organization of Economic Co-Operation and Development definition (OECD 2015). Participants had the option to report a specific amount or to select the appropriate range from a list. TwinLife also had three measures of occupation for each parent: the 2008 International Standard Classification of Occupations (International Labor Office 2012), the International Socio-Economic Index (ISEI; OECD, Education at a glance 2002; reported by offspring), and the Erikson–Goldthorpe–Portocarero Class Schedule (Erikson et al. 1979). We averaged the individual parental education measures across parents, and took the maximum across parents of each occupational index. Factor analysis of all six (including income) indicated that the ISEI functioned very differently from the others (factor loading 0.12 relative to > 0.70 for the other education and occupation measures), so we dropped it. We standardized each of the others, gave double weight to income and use the resulting average of the remaining five as our composite measure of SES.

MTFS had a single 12-level educational attainment scale, with levels ranging from high-school dropout through partial and complete trade school, associate degree, 4-year university degree, and levels of graduate and professional degrees. Parents reported pre-tax household income in ranges. Given the time span across which MTFS assessments were conducted, we adjusted estimated means within each range using the 1995 (midpoint of reporting period) Consumer Price Index. Parents who had full-time jobs reported occupational status using the Hollingshead (1957) Index. We averaged the education scale across parents, took the maximum occupational status across parents, standardized each of the resulting three (including income) measures, and used their mean as our composite measure of SES.

### Analytical approach

As a background check, we estimated and compared genetic (A) and shared (C) and non-shared (E) environmental variances in BMI separately in each sex in each TwinLife cohort and at each assessed age shared with TwinLife in MTFS. We estimated the sexes separately because BMI, overweight, and obesity rates and developmental patterns differ by sex, making differences in gene-environment interplay very likely. These estimates rely on the assumption that greater similarity of MZ than DZ twin pairs growing up together can be attributed to additive genetic influences independently of environmental influences that act to make members of both kinds of twin pairs similar and of environmental influences that act to make members of both kinds of twin pairs different, including measurement error. They also rely on the assumption that parents have mated randomly with respect to the traits of interest, and MZ and DZ twins are exposed to environments tending to make them similar to the same degree. Because parental data were available, we were able to estimate the effects on DZ twin genetic relatedness resulting from the observed parental correlations. Some studies have suggested non-additive as well as additive genetic influences on body weight (e.g., Stunkard et al. 1994), but both cannot be estimated simultaneously, and there was little evidence of non-additive influences in our data.

The univariate model we used can be extended to examine genetic and environmental inter-relations between two characteristics. This is accomplished by estimating the A, C, and E components of the covariance. In standardized form, these covariance components are termed genetic and shared and non-shared environmental correlations, and indicate the extent to which the same sources of influence are involved in both characteristics. Often such genetic correlations are interpreted as indicating that at least some of the underlying genes are pleiotropic—they directly control multiple otherwise unrelated biological pathways, potentially indicating absence of direct environmental effects. Genetic correlations can also arise due to population stratification, linkage disequilibrium, sampling bias, assortative mating, and other deviations from accurate measurement and/or fully representative sampling, but likely more importantly and more commonly, they arise over developmental time *whenever* almost inevitably genetically influenced environmental circumstances *do* have direct effects on other personal characteristics. This is because, as those genetically influenced circumstances exert their effects, the underlying genetic influences on them will contribute to apparent genetic variance in the other characteristic over time. This is also true of the specific circumstances underlying shared and non-shared environmental correlations. This makes genetic correlation as inevitable as presence of genetic influence on each characteristic whenever two characteristics are phenotypically correlated. It also makes these underlying–influence correlations as uninformative as phenotypic correlations about direction of causation.

**Fig. 1:**
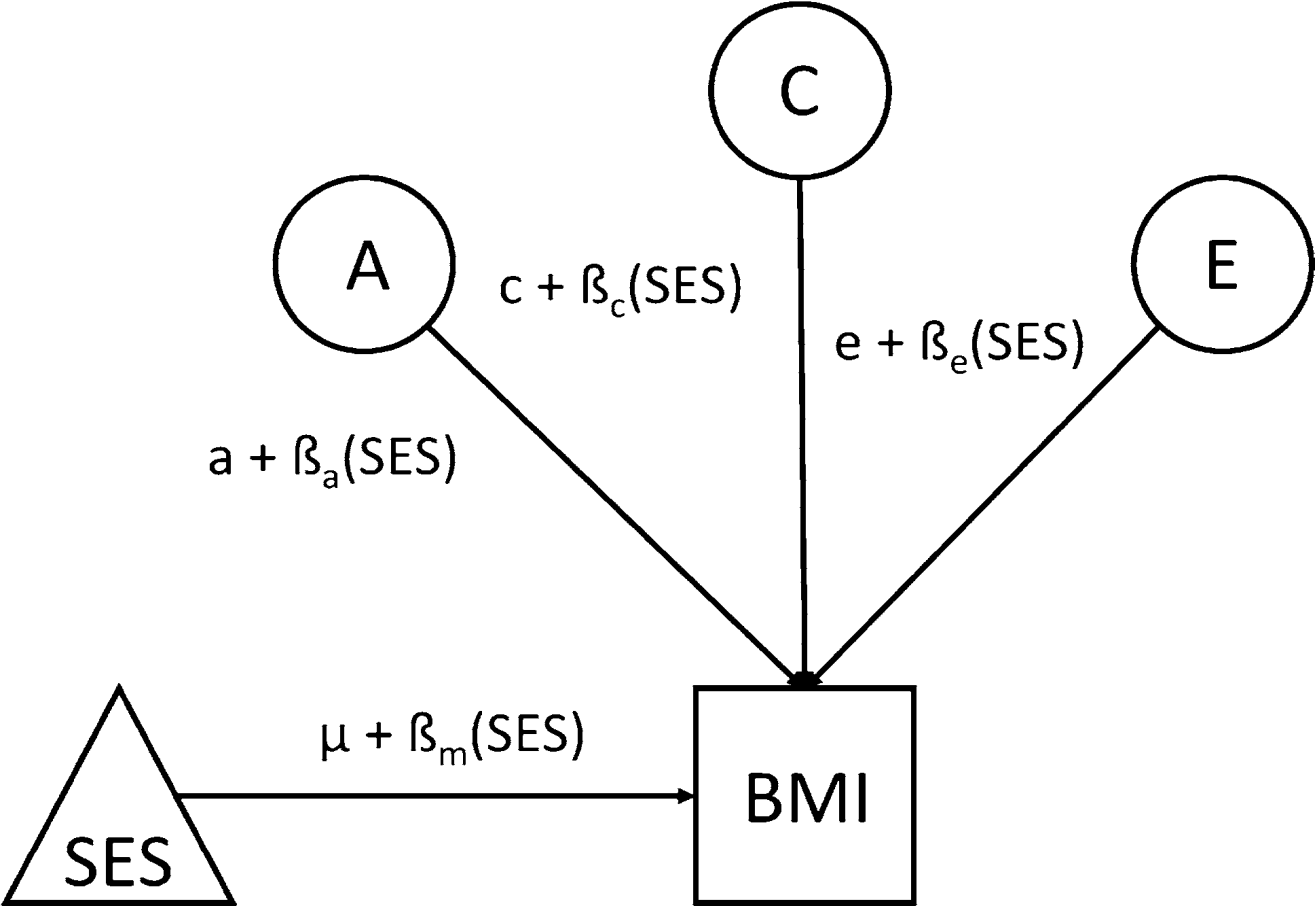
Model of socioeconomic status-of-origin moderating Body Mass Index in children and adolescents. A refers to genetic influences, C to shared environmental influences, and E to non-shared environmental influences

There is a way to extend this model, however, that can at least begin to hint at causal directions and pathways. This is done by relaxing the assumption that genetic and environmental influences operate independently, and, in particular, estimates extents to which genetic and environmental influences interact so that variance components in one variable vary with the phenotypic magnitude of the other (Purcell 2002). This is the model that was used to derive the observations cited in this article’s introduction that higher adult SES tends to be associated with both lower mean BMI levels and less genetic variance in BMI. As noted there, however, the model is more limited in situations where twin pairs share the environmental moderator, as is the case for SES-of-origin. Figure 1 depicts the model. In it, the triangle labelled SES reflects the confounded potential main effect of SES on BMI and their potentially moderated covariance. The latent A, C, and E variables reflect variance in BMI not shared with SES.

We implemented this model in Mx (Neale et al. 2003), using maximum likelihood estimation to include even cases with incomplete data. Because we were interested in comparing actual variances among groups, we report absolute variance components rather than proportions of variance unless otherwise noted. We checked that partitioning variables in equal scale intervals indicated no trends in interval variances. This is important in avoiding confounding of arbitrary measurement scale with variance moderation (Falconer and Mckay 1989). We tested the significance of each moderating and main effect parameter and dropped from presentation those that were not significant and whose absence did not reduce model fit. This was not to disregard their possible involvement, but rather to focus on the most important moderating parameters and to facilitate comparison of differences between countries, sexes, and age groups. We evaluated model fit based on significant difference in log-likelihood, Akaike’s Information Criterion (AIC; Akaike 1983), and Bayesian Information Criterion (BIC; Raftery 1995). We focused on moderating parameter significance as indicated by model fit rather than confidence intervals because estimating the latter is much less accurate when the genetic and environmental influences they reflect vary with the level of a moderator.

## Results and discussion

### Descriptive statistics

Table 2 shows descriptive statistics for the raw parental variables contributing to this article. Both cohort and age effects are visible in both studies, as well as differences between them. Parents of younger and more recently born twins were younger on average, and German parents tended to be older than Minnesotan parents, matched for twin ages. This likely reflected trends toward later parenting (OECD 2017), as the German parents tended to have been born about a generation later. Parents of younger and more recently born twins were also taller on average, reflecting world-wide general height trends over the past century (Clark 2008). German parents tended to be taller than Minnesota parents, likely also reflecting their more recent birth. Parents of younger twins tended to weigh less on average, consistent with generally observed age trends in weight in adulthood, and German parents tended to weigh noticeably less than Minnesotan parents, matched for twin ages, despite Minnesota parents being assessed on average about 20 years previously. This was reflected in the BMIs as well. German parents varied more in height, but Minnesotan parents varied more in weight and BMI. In both countries, fathers’ heights varied more than mothers’, but mothers’ weights, and especially BMIs, varied more than fathers’.

**Table 2:** Parental descriptive statistics

TwinLife	Mean	SD	Range	Mean	SD	Range	Mean	SD	Range	Mean	SD	Range
	Cohort											
	1—Age ~ 5 years			2—Age ~ 11 years			3—Age ~ 17 years			4—Age ~ 23–24 years		
Mother’s age	37.3	5.1	22–59	43.0	4.9	28–58	47.8	4.6	34–63	52.6	4.6	41–69
Mother’s height	168.0	6.7	143–195	168.3	6.4	148–188	167.7	6.4	150–189	166.5	6.6	126–186
Mother’s weight	70.1	15.0	39–149	71.3	14.6	39–150	71.9	15.0	40–150	72.6	14.8	41–158
Mother’s BMI	24.8	5.1	16.0–52.8	25.2	5.0	15.9–49.6	25.6	5.3	15.1–53.2	26.2	5.4	16.4–61.7
Father’s age	40.9	6.3	24–72	46.5	5.5	29–78	50.6	5.0	34–74	55.1	5.7	22–79
Father’s height	180.9	7.2	154–206	180.5	7.0	150–203	180.4	7.4	117–200	179.3	7.1	155–204
Father’s weight	86.9	14.1	55–150	87.9	14.8	52–170	89.3	15.0	55–182	88.6	14.3	55–186
Father’s BMI	26.5	3.9	17.3–44.8	26.9	4.2	15.3–49.1	27.4	4.2	20.2–57.7	27.6	4.2	15.7–54.9
Highest parent ISCO	43.7	21.8	3–96	44.6	22.4	11–96	46.0	22.4	11–96	47.5	23.2	11–96
Highest parent EGP	31.4	20.1	1–96	33.8	21.7	1–96	36.9	22.4	1–96	38.2	24.3	1–96
Mean parent ISCED	7.1	2.5	1–11	7.1	2.4	1–11	6.9	2.4	1–11	6.5	2.5	1–11
Mean parent Educ.	3.9	1.1	1–6	3.8	1.1	1–6	3.7	1.1	1–6	3.4	1.2	1–6
Net household Inc.	1072.1	1137.9	74–28571	1097.3	919.4	70–13953	1067.0	700.1	82–5714	1384.8	901.4	41–10000

MTFS	Cohort											
	11-YO			17-YO								
Mother’s age	40.2	4.9	28–56	44.3	4.7	33–60						
Mother’s height	165.2	5.9	140–198	163.8	5.9	143–180						
Mother’s weight	75.9	19.1	42–154	77.0	19.2	47–159						
Mother’s BMI	27.8	6.8	17–58	28.7	6.9	18–57						
Father’s age	42.6	5.4	29–65	46.6	5.4	32–66						
Father’s height	178.1	6.9	133–202	177.2	6.4	152–197						
Father’s Weight	91.2	17.7	53–184	91.1	15.0	54–147						
Father’s BMI	28.7	5.1	15.5–52.6	29.0	4.5	18.3–48.1						
Highest parent Occ.	4.9	1.5	1–7	4.7	1.6	1–7						
Mean parent Educ.	4.5	2.6	0–9	3.9	2.6	0–9						
Household income	50.8	22.8	7–102	53.0	23.8	7–102						

*BMI* Body Mass Index. Heights are in cm., weights in kg. *ISCO* International Standard Class of Occupations, 2008. *EGP* Erikson–Goldthorpe–Portocarero Class Scheme. *ISCED* International Standard Classification of Education, 1997. TwinLife Net Household Income is based on Organization for Economic Development and Cooperation coding scheme. MTFS Occupation is reversed Hollingshead Scale, Education is based on a 10-point scale ranging from less than high school to PhD/professional degree. MTFS Household Income developed from means of categorical ranges adjusted from year reported to 1995 (median reporting year) level, in thousands of dollars. TwinLife cohorts based on pair year of birth, MTFS cohorts based on twin-pair age at recruitment

**Fig. 2:**
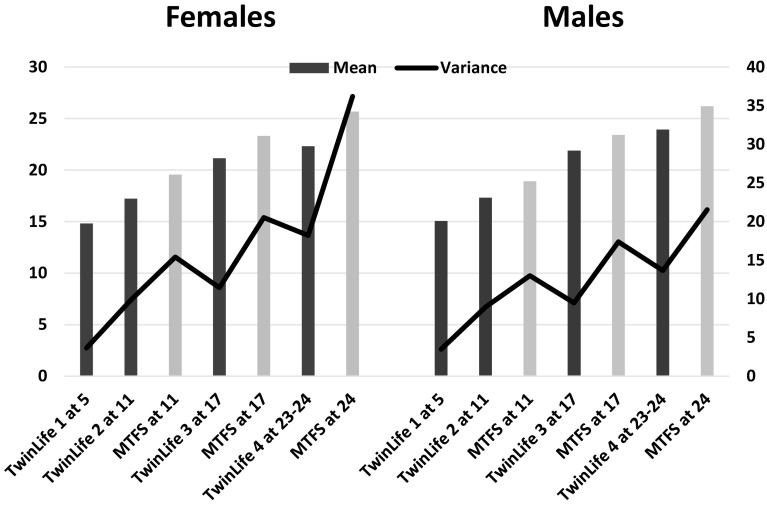
BMI means and variances in female and male twins from TwinLife in Germany and MTFS in Minnesota. Bars refer to mean levels, lines to variances. All country differences were significant in both sexes. Digits for samples refer to ages

The raw twin descriptive statistics contributing to BMI are shown in Table 3. At age 11, Minnesota twins were taller, but this had reversed by age 17, suggesting earlier puberty-related growth spurts in Minnesota, especially for girls. Minnesota twins weighed on average as much as 1 standard deviation more at all ages; this was consequently reflected in higher BMIs. At age 11, Minnesota girls were slightly taller and weighed more than boys (consistent with girls’ earlier puberty), but they were evenly matched in Germany, and boys were taller and weighed considerably more in both countries by age 17 (slightly taller and heavier at age 5 too). Variances in height were greatest at age 11, reflecting variance in timing of the pubertal growth spurt, but variances in weight and BMI increased with age in both sexes and countries. Like their parents, German twins varied more in height than did Minnesota twins, but Minnesota twins varied more in weight and BMI. Except at age 11, boys resembled their fathers in varying more in height than did girls. Minnesota girls resembled their mothers in varying more in weight and BMI than boys at all ages, but German girls only showed greater variance in BMI in Cohort 4 (ages 23–24). Figure 2 summarizes these comparisons in graphical form. The means were consistent with data from other, larger sources, but these sources do not usually address variances. Differences in variance may indicate factors relevant to mean level trends, however, such as society-level disparities in economic and community resources.

**Table 3:** Twin descriptive statistics

	Mean	SD	Range	Mean	SD	Range	Mean	SD	Range	Mean	SD	Range
TwinLife												
Cohort	1—Age ~ 5 years			2—Age ~ 11 years			3—Age ~ 17 years			4—Age 23–24 years		
Females												
Pair age (mos.)	65.2	3.9	58–77	137.5	3.7	130–147	209.6	3.9	201–220	282.1	9.4	261–303
Twin 1 height	113.1	5.6	95–128	149.6	8.3	124–176	167.1	7.0	100–184	166.7	6.8	145–188
Twin 2 height	113.1	6.0	97–135	149.6	8.2	124–176	166.9	6.4	149–184	166.8	7.6	115–188
Twin 1 weight	19.1	3.0	11–34	38.8	9.3	24–106	59.1	10.0	38–130	62.4	13.4	38–140
Twin 2 weight	19.1	3.3	12–39	38.9	10.6	21–160	58.6	9.6	33–130	62.2	13.3	39–144
Twin 1 BMI	14.8	1.8	10–28	17.2	3.1	12–34	21.2	3.2	15–39	22.4	4.2	15–55
Twin 2 BMI	14.8	2.0	10–37	17.2	3.2	11–35	21.1	3.1	13–39	22.2	4.3	15–46
Males												
Pair age (mos.)	65.7	3.9	52–75	137.5	3.7	130–147	209.8	3.9	201–220	281.6	10.1	210–304
Twin 1 height	113.6	6.1	90–145	149.8	8.2	80–172	180.5	7.3	156–201	181.1	7.2	155–200
Twin 2 height	113.4	6.4	80–143	149.4	8.7	80–172	180.6	7.0	156–199	180.1	7.6	146–204
Twin 1 weight	19.5	3.2	12–34	39.3	8.4	22–78	71.7	11.1	36–126	78.3	13.8	47–144
Twin 2 weight	19.4	3.2	13–35	38.7	8.3	22–75	71.3	11.9	42–13	78.3	14.0	50–139
Twin 1 BMI	15.1	1.8	11–24	17.4	3.0	10–35	22.0	3.0	15–37	23.8	3.7	15–43
Twin 2 BMI	15.0	1.9	11–26	17.3	2.9	8–35	21.8	3.1	15–38	24.1	3.7	16–42

MTFS												
Assessment age				11 years (TL Cohort 2)			17 years (TL Cohort 3)			24 years (TL Cohort 4)		
Females												
Twin 1 age (mos.)				141.3	5.6	129–156	213.3	7.7	199–261	300.6	7.0	286–335
Twin 2 age (mos.)				141.3	5.6	129–156	213.3	7.9	199–272	300.6	7.0	286–335
Twin 1 height				151.7	7.6	126–174	164.9	6.3	149–184	166.3	6.5	148–182
Twin 2 height				151.3	7.4	127–173	164.8	6.4	147–184	166.1	6.4	150–184
Twin 1 weight				45.9	11.7	25–98	64.0	13.8	40–149	72.1	19.3	38–155
Twin 2 weight				44.8	11.8	25–107	63.1	13.7	39–141	70.3	17.4	42–161
Twin 1 BMI				19.7	3.9	14–35	23.5	4.5	16–45	25.9	6.3	16–50
Twin 2 BMI				19.4	3.9	13–38	23.2	4.5	16–45	25.4	5.7	16–51
Males												
Twin 1 age (mos.)				141.4	4.8	129–155	212.7	6.8	198–274	296.8	11.6	272–349
Twin 2 age (mos.)				141.4	4.8	128–176	212.6	6.3	198–245	296.7	11.5	272–349
Twin 1 height				150.2	7.6	127–179	177.8	6.7	159–202	178.9	6.6	162–203
Twin 2 height				150.1	7.6	128–176	177.6	6.8	155–201	178.8	6.7	162–202
Twin 1 weight				43.4	10.9	26–122	74.4	14.5	46–141	84.7	16.7	55–158
Twin 2 weight				42.6	10.6	25–108	73.6	13.7	41–148	83.0	15.3	45–143
Twin 1 BMI				19.1	3.7	12–38	23.5	4.3	17–45	26.5	4.9	18–51
Twin 2 BMI				18.8	3.5	14–38	23.2	4.0	15–43	25.9	4.4	17–46

Ages in months. BMI is Body Mass Index. Heights in cm., weights in kg. No significant differences between Twin 1 and Twin 2 in height, weight, or BMI after within-cohort, within-sex age-adjustment in Twin-Life, within-sex age-adjustment in MTFS. MTFS age data for recruitment-age cohorts where available (ages 17 and 24, selected to match TwinLife cohort ages as well as possible (TwinLife analog in parentheses). TwinLife families were assessed always assessed together; MTFS often but not always

### Rates of overweight and obesity

Table 4 shows rates of overweight and obesity in parents and twins, based on the standard BMI cut-offs of 25 and 30 for adults and both CDC and WHO standards for those under age 18. Parental rates were generally consistent with WHO and CDC statistics in magnitude, with Minnesotan rates considerably higher than German rates, especially among mothers. In contrast to WHO (2017) data, however, overweight rates were lower in mothers than in fathers in both countries, and obesity rates in Germany were lower too, though not in Minnesota. This could reflect the slightly elevated overall levels of education in both samples, especially if associated with greater attention to maintenance of muscle mass (physical fitness) in adulthood. MTFS age-24 and TwinLife Cohort 4 (ages 23–24) rates ran well behind their parents’, but were considerably higher than those of the younger twins (including, in MTFS, themselves at younger ages). The rates in younger twins showed likely impact of greater weight of muscle than fat mass in young males, which distorts BMI as a measure of adiposity. Overall, however, the rates were very consistent with concerns about indications that childhood overweight and obesity are emerging at younger ages and to increasing degrees over time, more so in the United States than in Germany.

**Table 4:** Rates of overweight and obesity

	Adult standards			Child and adolescent standards			
	% BMI ≥ 25	% BMI ≥ 30	Mean BMI in the obese	% Over-weight WHO	% Over-weight CDC	% obese WHO	% obese CDC
TwinLife							
Mothers	41.9	16.2	–	–	–	–	–
Fathers	66.4	18.0	–	–	–	–	–
Female twins							
1. Age ~ 5 years	–	–	19.9	7.4	7.4	2.0	4.1
2. Age ~ 11 years	–	–	25.9	16.0	11.3	3.9	3.8
3. Age ~ 17 years	–	–	30.7	11.7	9.5	2.2	2.4
4. Age 23–24 years	16.1	5.3	–	–	–	–	–
Male twins							
1. Age ~ 5 years	–	–	20.2	11.1	11.1	5.3	6.1
2. Age ~ 11 years	–	–	25.3	47.0	16.7	7.1	4.6
3. Age ~ 17 years	–	–	30.5	14.4	12.1	2.8	3.6
4. Age 23–24 years	27.5	7.5	–	–	–	–	–
MTFS							
Mothers	57.7	30.7	36.4	–	–	–	---
Fathers	66.9	27.9	34.2	–	–	–	–
Female twins							
Age 11	–	–	27.4	35.1	26.4	13.2	13.3
Age 17	–	–	34.9	23.1	23.1	8.4	9.1
Age 24	43.5	19.2	35.7	–	–	–	–
Male twins							
Age 11	N/A	N/A	26.1	34.3	18.6	13.8	12.5
Age 17	N/A	N/A	33.8	27.7	23.8	8.6	10.1
Age 24	53.1	16.9	33.3	N/A	N/A	N/A	N/A

For adults BMI ≥ 25 is considered overweight, ≥ 30 obese. For children and teens, WHO standard for overweight is 1 standard deviation above the mean for age in their reference sample, and that for obesity is 2 standard deviations above that mean for age. Mean BMI in the obese was calculated in our samples using the WHO standards. The CDC standard for overweight in children and teens is the 85th percentile for age in their reference sample, and that for obesity is the 95th percentile. See text for further details on these standards

*BMI* Body Mass Index, *SD* standard deviation. Overweight rates include the obese

### Phenotypic correlations

**Fig. 3:**
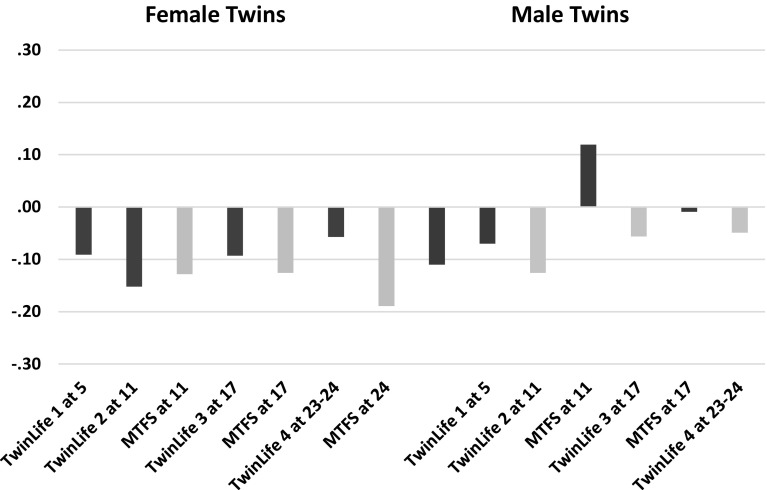
Phenotypic correlations of BMI with SES in female and male twins from TwinLife in Germany and MTFS in Minnesota. Digits for samples refer to ages

Tables 5 and 6 show the phenotypic correlations among study variables, Table 5 for TwinLife and Table 6 for MTFS. Reflecting accumulation of overweight with age, correlations between height and weight were generally larger in younger groups and small in adulthood. Parental assortment for height was generally moderate, but that for weight and BMI small. There was enough parental assortment on BMI, however, to indicate that, on average, DZ twins would share 55% of their segregating genes rather than 50% when parental assortment is random (parental correlations 0.23 in TwinLife, 0.22 in MTFS). We adapted our modelling assumptions to reflect this. SES was generally associated positively with height and negatively with weight and BMI to small degrees, in both parents and twins in all age groups. One exception to this was TwinLife Cohort 3 (age 17) boys, for whom the SES–BMI correlation was positive. Because the phenotypic SES–BMI correlations were fundamental to our SES-moderation hypotheses, we highlight them in Fig. 3. Parent–offspring correlations reflected both genetic and family lifestyle transmission. Height correlations were moderate, and tended to be somewhat higher at older than younger ages and perhaps higher with the parent of the same sex than the opposite sex. Parent–offspring weight and BMI correlations were generally small to moderate and showed the same age pattern, but no particular pattern regarding same- and opposite-sex. Mid-parent–offspring correlations (not shown) were higher, but still ran about 0.10 lower than DZ correlations. These patterns were rather consistent in the two samples. They likely reflect combinations of gene-environment correlation for tendency to weight gain and lifestyle, generation-specific lifestyle effects, and development processes.

**Table 5:** TwinLife phenotypic correlations among study variables—by cohort and sex

	1	2	3	4	5	6	7	8	9	10
Cohort 1—Age ~ 5										
1. Mother’s Hgt.	1.000	0.316	− 0.048	0.294	0.146	− 0.012	0.388	0.293	0.049	0.134
2. Mother’s Wgt.	0.294	1.000	0.930	0.067	0.199	0.181	0.141	0.159	0.150	− 0.092
3. Mother’s BMI	− 0.101	0.918	1.000	− 0.040	0.163	0.204	0.004	0.054	0.090	− 0.145
4. Father’s Hgt.	0.240	0.006	− 0.083	1.000	0.401	− 0.112	0.361	0.217	− 0.042	0.218
5. Father’s Wgt.	0.047	0.145	0.129	0.464	1.000	0.862	0.155	0.186	0.130	− 0.029
6. Father’s BMI	− 0.082	0.154	0.187	− 0.032	0.867	1.000	− 0.031	0.084	0.164	− 0.154
7. Twin Hgt.	0.373	0.153	0.002	0.407	0.215	0.010	1.000	0.620	− 0.040	0.123
8. Twin Wgt.	0.143	0.142	0.088	0.285	0.223	0.093	0.614	1.000	0.749	− 0.038
9. Twin BMI	− 0.099	0.052	0.101	0.010	0.124	0.146	0.003	0.775	1.000	− 0.110
10. Family SES	0.094	− 0.141	− 0.183	0.249	0.031	− 0.107	0.164	− 0.025	− 0.091	1.000
Cohort 2—Age ~ 11										
1. Mother’s Hgt.	1.000	0.246	− 1.500	0.207	0.140	0.041	0.363	0.179	0.007	0.077
2. Mother’s Wgt.	0.324	1.000	0.918	0.066	0.276	0.263	0.225	0.281	0.208	− 0.157
3. Mother’s BMI	− 0.045	0.927	1.000	− 0.020	0.225	0.255	0.078	0.216	0.210	− 0.199
4. Father’s Hgt.	0.306	0.087	− 0.030	1.000	0.432	− 0.055	0.284	0.129	− 0.004	0.124
5. Father’s Wgt.	− 0.036	0.169	0.206	0.397	1.000	0.874	0.258	0.264	0.167	0.052
6. Father’s BMI	− 0.185	0.143	0.240	− 0.062	0.888	1.000	0.129	0.207	0.171	− 0.050
7. Twin Hgt	0.342	0.224	0.108	0.304	0.145	0.009	1.000	0.604	0.138	0.054
8. Twin Wgt.	0.182	0.319	0.269	0.151	0.281	0.231	0.630	1.000	0.864	− 0.044
9. Twin BMI	0.028	0.280	0.286	− 0.014	0.281	0.308	0.191	0.874	1.000	− 0.070
10. Family SES	0.086	− 0.080	− 0.117	0.067	− 0.052	− 0.093	0.042	− 0.096	− 0.152	1.000
Cohort 3—Age ~ 17										
1. Mother’s Hgt.	1.000	0.259	− 0.106	0.211	0.024	− 0.084	0.471	0.220	0.002	0.038
2. Mother’s Wgt.	0.216	1.000	0.930	− 0.062	0.141	0.203	0.151	0.279	0.252	− 0.165
3. Mother’s BMI	− 0.171	0.922	1.000	− 0.142	0.135	0.240	− 0.021	0.201	0.256	− 0.194
4. Father’s Hgt.	0.342	0.131	− 0.004	1.000	0.486	− 0.008	0.389	0.165	− 0.054	0.171
5. Father’s Wgt.	0.122	0.224	0.182	0.439	1.000	0.852	0.209	0.271	0.175	0.012
6. Father’s BMI	− 0.052	0.180	0.208	− 0.037	0.840	1.000	0.017	0.247	0.268	− 0.068
7. Twin Hgt.	0.488	0.055	− 0.133	0.505	0.233	− 0.024	1.000	0.484	− 0.070	0.208
8. Twin Wgt.	0.287	0.233	0.118	0.222	0.255	0.117	0.432	1.000	0.867	− 0.033
9. Twin BMI	0.049	0.208	0.185	− 0.047	0.152	0.144	− 0.062	0.866	1.000	0.119
10. Family SES	0.043	0.148	− 0.169	0.163	− 0.059	− 0.145	0.086	− 0.022	− 0.093	1.000
Cohort 4—Ages 23–24										
1. Mother’s Hgt.	1.000	0.196	− 0.200	0.265	0.260	0.125	0.436	0.241	0.450	0.172
2. Mother’s Wgt.	0.281	1.000	0.913	− 0.109	0.315	0.391	0.095	0.197	0.167	− 0.073
3. Mother’s BMI	− 0.120	0.915	1.000	− 0.223	0.223	0.358	− 0.062	0.112	0.155	− 0.135
4. Father’s Hgt.	0.309	0.082	− 0.054	1.000	0.365	− 0.133	0.459	0.121	− 0.116	0.212
5. Father’s Wgt.	0.199	0.219	0.146	0.409	1.000	0.871	0.268	0.337	0.228	0.067
6. Father’s BMI	0.044	0.191	0.187	− 0.131	0.847	1.000	0.034	0.287	0.300	− 0.035
7. Twin Hgt.	0.514	0.167	− 0.041	0.526	0.262	− 0.017	1.000	0.506	0.060	0.154
8. Twin Wgt.	0.202	0.315	0.233	0.244	0.311	0.201	0.418	1.000	0.888	0.071
9. Twin BMI	− 0.014	0.279	0.283	0.020	0.215	0.224	− 0.018	0.911	1.000	− 0.009
10. Family SES	0.148	− 0.030	− 0.102	0.206	0.048	− 0.059	0.121	− 0.020	− 0.057	1.000

*BMI*Body Mass Index, *SES* socioeconomic status. Most correlations stronger than 0.06 significant at *p* < 0.05, no adjustment for multiple testing. As a generality, when the larger correlation was 0.3 or less, difference between correlations of .15 was required for significance; above that, difference of .10 sufficed. Males twins above diagonals, females below. All height, weight, and BMI data age-adjusted within cohort, separately by family member

**Table 6:** MTFS phenotypic correlations among study variables—by assessment age and sex

	1	2	3	4	5	6	7	8	9	10
Age-11 Assessment										
1. Mother’s Hgt.	1.000	0.242	− 0.034	0.150	0.098	0.034	0.377	0.140	− 0.015	0.072
2. Mother’s Wgt.	0.219	1.000	0.959	0.012	0.215	0.233	0.162	0.316	0.313	− 0.183
3. Mother’s BMI	− 0.067	0.956	1.000	− 0.032	0.191	0.227	0.063	0.293	0.334	− 0.206
4. Father’s Hgt.	0.232	0.088	0.024	1.000	0.412	− 0.010	0.384	0.163	− 0.006	0.089
5. Father’s Wgt.	0.170	0.243	0.200	0.405	1.000	0.903	0.257	0.305	0.243	− 0.054
6. Father’s BMI	0.087	0.225	0.205	0.005	0.910	1.000	0.113	0.270	0.280	− 0.097
7. Twin Hgt.	0.342	0.197	0.097	0.403	0.273	0.131	1.000	0.617	0.265	0.034
8. Twin Wgt.	0.109	0.360	0.330	0.139	0.334	0.308	0.654	1.000	0.918	− 0.088
9. Twin BMI	− 0.021	0.353	0.362	− 0.026	0.283	0.320	0.367	0.940	1.000	− 0.126
10. Family SES	0.146	− 0.180	− 0.226	0.134	− 0.016	− 0.075	0.092	− 0.071	− 0.128	1.000
Age-17 Assessment										
1. Mother’s Hgt	1.000	0.242	− 0.034	0.150	0.098	0.034	0.434	0.166	− 0.006	0.072
2. Mother’s Wgt.	0.219	1.000	0.959	0.012	0.215	0.233	0.124	0.379	0.355	− 0.183
3. Mother’s BMI	− 0.067	0.956	1.000	− 0.032	0.191	0.227	0.002	0.342	0.368	− 0.206
4. Father’s Hgt.	0.232	0.088	0.024	1.000	0.412	− 0.010	0.457	0.158	− 0.041	0.089
5. Father’s Wgt.	0.170	0.243	0.200	0.405	1.000	0.903	0.236	0.349	0.273	− 0.054
6. Father’s BMI	0.087	0.225	0.205	0.005	0.910	1.000	0.046	0.316	0.329	− 0.097
7. Twin Hgt.	0.484	0.143	0.009	0.528	0.280	0.089	1.000	0.354	− 0.057	0.070
8. Twin Wgt.	0.154	0.352	0.315	0.205	0.353	0.304	0.383	1.000	0.910	− 0.020
9. Twin BMI	− 0.025	0.318	0.332	0.007	0.266	0.291	0.016	0.926	1.000	− 0.056
10. Family SES	0.146	− 0.180	− 0.226	0.134	− 0.016	− 0.075	0.112	− 0.074	− 0.126	1.000
Age-24 Assessment										
1. Mother’s Hgt.	1.000	0.242	− 0.034	0.150	0.098	0.034	0.411	0.143	− 0.010	0.072
2. Mother’s Wgt.	0.219	1.000	0.959	0.012	0.215	0.233	0.159	0.366	0.328	− 0.183
3. Mother’s BMI	− 0.067	0.956	1.000	− 0.032	0.191	0.227	0.043	0.333	0.340	− 0.206
4. Father’s Hgt.	0.232	0.088	0.024	1.000	0.412	− 0.010	0.448	0.172	− 0.012	0.089
5. Father’s Wgt.	0.170	0.243	0.200	0.405	1.000	0.903	0.247	0.358	0.281	− 0.054
6. Father’s BMI	0.087	0.225	0.205	0.005	0.910	1.000	0.055	0.319	0.325	− 0.097
7. Twin Hgt.	0.510	0.198	0.054	0.630	0.322	0.095	1.000	0.372	− 0.018	0.036
8. Twin Wgt.	0.156	0.389	0.345	0.250	0.298	0.235	0.399	1.000	0.918	− 0.030
9. Twin BMI	0.010	0.356	0.353	0.061	0.221	0.231	0.104	0.950	1.000	− 0.049
10. Family SES	0.146	− 0.180	− 0.226	0.134	− 0.016	− 0.075	0.139	− 0.132	− 0.189	1.000

MTFS twin cross-time cross-trait correlations										
	1	2	3	4	5	6	7	8	9	
1. Age-11 Height	1.000	0.617	0.265	0.747	0.427	0.149	0.717	0.451	0.184	
2. Age-11 Weight	0.654	1.000	0.918	0.267	0.781	0.718	0.292	0.693	0.617	
3. Age-11 BMI	0.367	0.940	1.000	− 0.039	0.743	0.809	0.012	0.650	0.690	
4. Age-17 Height	0.681	0.269	0.014	1.000	0.354	− 0.057	0.971	0.366	− 0.007	
5. Age-17 Weight	0.442	0.755	0.723	0.383	1.000	0.910	0.375	0.823	0.729	
6. Age-17 BMI	0.220	0.706	0.769	0.016	0.926	1.000	− 0.009	0.731	0.794	
7. Age-24 Height	0.729	0.331	0.070	0.989	0.410	0.056	1.000	0.372	− 0.018	
8. Age-24 Weight	0.468	0.727	0.689	0.375	0.822	0.750	0.399	1.000	0.918	
9. Age-24 BMI	0.274	0.682	0.728	0.064	0.739	0.790	0.104	0.950	1.000	

*BMI* Body Mass Index, *SES* socioeconomic status. Most correlations stronger than 0.04 significant at *p* < 0.05, no adjustment for multiple testing. As a generality, when the larger correlation was 0.3 or less, difference between correlations of 0.15 was required for significance; above that, difference of 0.10 sufficed. Males twins above diagonals, females below. All height, weight, and BMI data age- and recruitment year-adjusted, separately by family member. Cohorts combined as available; parents assessed at first twin assessment

In MTFS, cross–time correlations of height were 0.60–0.80 before age 17, and well above 0.90 after that. Weight and BMI cross–time correlations were similar at the younger ages, but not as high after age 17. Girls tended to be slightly more consistent across time than boys.

### Univariate variance decompositions

**Fig. 4:**
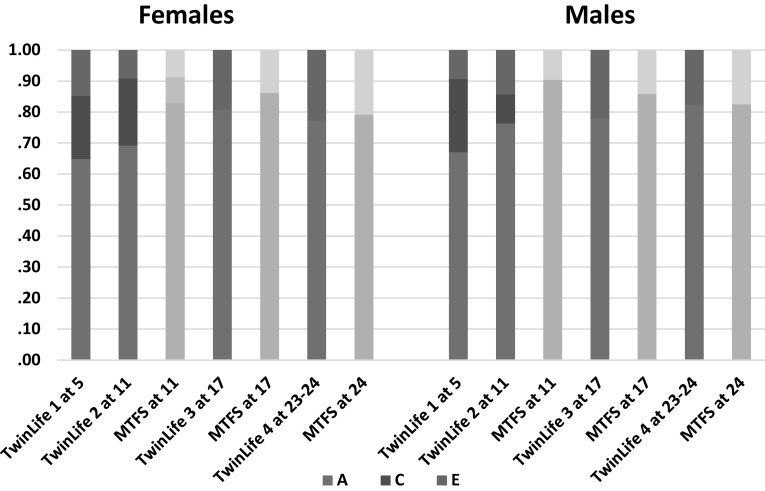
Basic estimates of proportions of variance in BMI attributable to genetic (A) and shared (C) and non-shared environmental influences in female and male twins from TwinLife in Germany and MTFS in Minnesota. Digits for samples refer to ages

The twin correlations are shown in Table 7. Presence of genetic influence on height, weight, and BMI was clear throughout. Except at age 11 in MTFS girls and in TwinLife Cohort 4 (ages 23–24) men, all indicated important shared environmental influences on height as well. At the younger ages, weight and BMI also showed important shared environmental influences, but these eroded in the older ages. We fit standard univariate twin models to these data to produce the estimates of proportions of variance in BMI attributable to genetic and shared and non-shared environmental influences shown in Fig. 4. At all ages in both sexes in both countries, the majority of the variance could be attributed to genetic influence.

**Table 7:** Twin correlations

TwinLife	MZ	DZ	MZ	DZ	MZ	DZ	MZ	DZ
Cohort	1—Age ~ 5		2—Age ~ 11		3—Age ~ 17		4—Ages 23–24	
Females								
Height	0.932	0.632	0.928	0.657	0.897	0.520	0.901	0.557
Weight	0.863	0.568	0.922	0.595	0.805	0.377	0.812	0.365
BMI	0.866	0.542	0.905	0.594	0.775	0.370	0.786	0.312
Males								
Height	0.910	0.597	0.898	0.517	0.879	0.580	0.902	0.421
Weight	0.922	0.518	0.890	0.524	0.829	0.377	0.862	0.509
BMI	0.913	0.584	0.875	0.497	0.780	0.338	0.832	0.419

MTFS								
Assessment age			11		17		24	
Females								
Height			0.930	0.441	0.929	0.534	0.945	0.624
Weight			0.907	0.545	0.857	0.443	0.819	0.428
BMI			0.902	0.582	0.836	0.420	0.800	0.416
Males								
Height			0.927	0.488	0.926	0.429	0.909	0.490
Weight			0.889	0.520	0.844	0.492	0.817	0.437
BMI			0.881	0.557	0.840	0.504	0.820	0.379

*BMI* Body Mass Index. All height, weight, and BMI age- and recruitment year-adjusted, MTFS cohorts combined as available

### SES-moderation models

We next fit the SES moderation models in each TwinLife cohort and at each assessment age in MTFS, separately in females and males. The second column of Table 8 indicates the best-fitting model in each (fit statistics leading to selection of best-fitting models and confidence intervals for their moderating parameters are provided in online supplementary information). With the exception of men in TwinLife Cohort 4 (ages 23–24), there were significant negative main effects of SES on BMI in all groups in both sexes and both countries, so that BMIs tended to be lower in youth of higher SES-of-origin. The effect was significantly positive in this one older male German group, however. [But it was TwinLife male Cohort 3 (age 17) that showed the lone positive phenotypic correlation.] In most groups, SES moderated the genetic influences on BMI, so that there was less genetic variance and heritability in BMI at higher levels of SES, consistent with the adult studies based on attained SES reviewed above. TwinLife Cohort 1 (age 5) girls and boys and TwinLife Cohort 3 boys (age 17) were exceptions to this. In these groups, there was less variance in BMI at higher levels of SES too, but instead of moderating genetic influences, SES moderated shared environmental influences. SES also moderated non-shared environmental influences in many groups. Usually this also meant less non-shared environmental variance at higher levels of SES, but it meant more non-shared environmental variance in MTFS males at ages 17 and 23, and TwinLife males in Cohorts 2 (age 11) and 4 (ages 23–24).

**Table 8:** Comparison of best-fitting moderation models

	Best-fitting individual model	Fit to other country’s parameters?	Constrain moderation equal?	Constrain moderation & mean equal?	Nature of difference
Females					
TwinLife 1—Germany	Mod. −A, −C, −B	N/A	–	–	–
TwinLife 2—Germany	Mod. −C, −E, −B	Yes	Mixed indications	Mixed indications	C var., mod., main eff. size
*MTFS 11—USA	Mod. −A, −E, −B	No			
TwinLife 3—Germany	Mod. −A, −B	Yes	Yes	Yes	None significant
*MTFS 17—USA	Mod. −A, −B	Yes			
*TwinLife 4—Germany	Mod. −A, −E, −B	No	Mixed indications	No	E mod., main eff. size
MTFS 24—USA	Mod. −A, −B	No			
Males					
TwinLife 1—Germany	Mod. −C, −E, −B	–	–	–	
TwinLife 2—Germany	Mod. +E, −B	Yes	Yes	Yes	None significant
*MTFS 11—USA	Mod. −A, −B	No			
TwinLife 3—Germany	Mod. −C, −E, −B	No	Mixed indications	Mixed indications	A, C Mod., main eff. size
*MTFS 17—USA	Mod. −A, +E, −B	No			
TwinLife 4—Germany	Mod. −A, +E, +B	Maybe	Yes	Yes	Mod. extent, main eff. dir.
MTFS 24—USA	Mod −A, +E, −B	No			

TwinLife numbers refer to birth cohorts, associated with assessment ages. MTFS numbers refer to assessment ages. Mod A, C, or E refers to significant parameters moderating genetic, shared, and non-shared environmental variance components, respectively, with sign indicating direction. ± B refers to significant positive or negative main effect at higher SES. In direct comparisons, * samples had greater power

We compared results in analogous age groups across countries in two ways. Our strictest test of differences was to fit each age–sex group’s data to the parameters generated in the analogous model of the other country’s data [e.g., by fixing the moderating and main-effect parameters to those generated by the girls’ age-11 MTFS data to TwinLife Cohort 2 (age 11) girls’ data]. In doing this, we allowed the A, C, and E parameters that reflect the magnitudes of the total variances to remain free, as it was plain that total variance in BMI was greater in MTFS than in TwinLife at all ages and in their parents as well. The extent to which we were able to do this depended not just on how different the parameters were, but on the relative sizes of the samples. In most groups, the power advantage went to MTFS, so there were several German groups in which we were able to do this without loss of model fit, but could not do it in the analogous Minnesotan groups.

To address which specific differences were most important, we also tested whether each significant moderating parameter could be constrained equal across countries in each group. This was possible without loss of model fit in girls at age 17 and in males at ages 11 and 24. In the other groups, some moderating parameters could be constrained equal, but not all. Maintaining equality constraints on the moderating parameters meriting it, we added equality constraints on the main effect parameters to each age–sex group’s two-country model. It was possible to do this in the three groups noted above without loss of fit, but not in the other three. Columns 3–5 in Table 8 summarize these results.

**Fig. 5:**
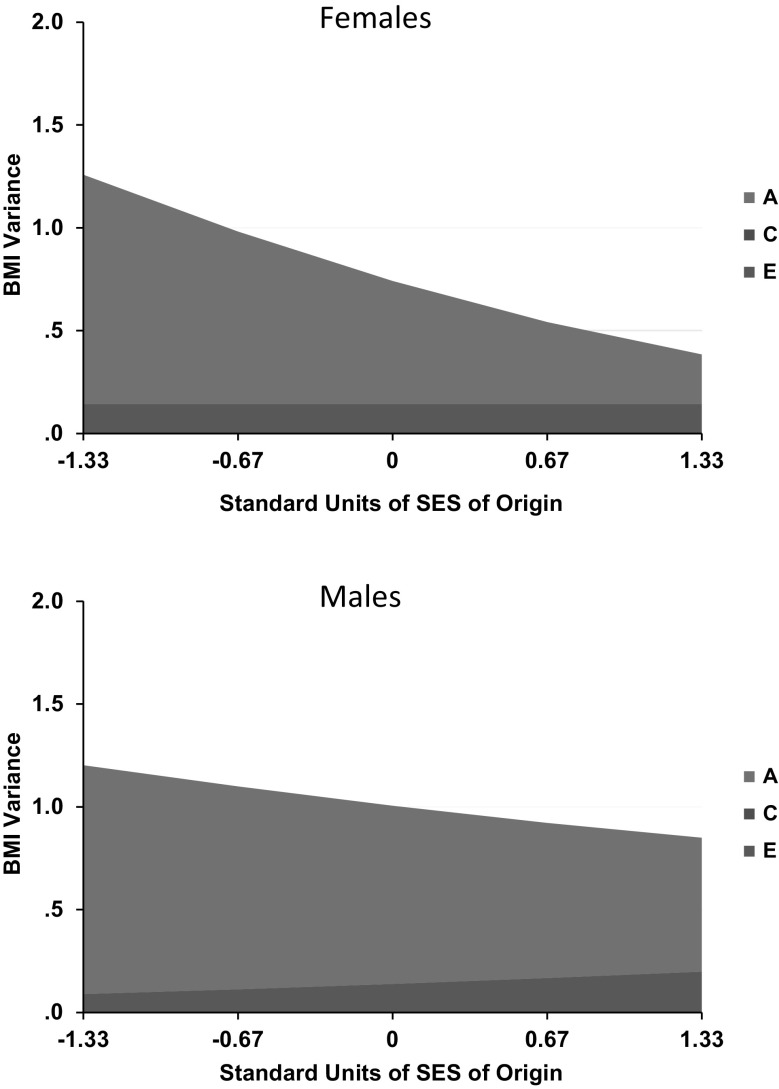
Most typical patterns of BMI genetic (A) and shared (C) and non-shared environmental variance moderation patterns by SES-of-origin in female and male twins from TwinLife in Germany and MTFS in Minnesota. In both sexes, these were specifically 17-year-old twins, and the best-fitting models constrained the moderating parameters but not the variance component patterns equal in the German and Minnesota samples

There was thus considerable consistency in results across age groups, countries, and sexes, as well as with observations in adults based on attained SES. Figure 5 shows the most typical patterns in the best-fitting models in each sex. At the same time, there was evidence of specific differences that suggested developmental patterns, sex differences, and country-specific contexts. Patterns in the female data were more consistent across both ages and countries. There was evidence of C moderation in TwinLife but not in MTFS, and it was consistent in direction with the observed A moderation wherever it appeared. Power to distinguish A variance from C variance is always relatively limited, and this is especially the case when the assumption that they are independent is violated. In German girls, evidence of C moderation was confined to the two younger cohorts, suggesting gene-environment correlation involving SES, genetic tendency to accumulate weight, and family lifestyles involving higher caloric intake, less exercise, and/or metabolic responses to stress in lower-SES groups. The parental lifestyle influence appeared to dissipate with age in girls, likely as they started to make more of their own diet and exercise choices, as well as choices involving experienced stress. In German boys, the C moderation appeared at ages 5 and 17. This suggested that parental influence might dissipate more rapidly in boys, but be replaced by within-pair peer- and school-related lifestyle choices such as similar levels of sports participation that affect muscle and thus weight development (without indicating obesity) to greater degrees in young males than in young females. The trends suggesting dissipation of parental influence were stronger at higher levels of SES.

Different emphasis on and opportunities for youth sports participation in Germany and the US, and their relations with SES may well have contributed to the TwinLife/MTFS differences observed. In addition to community-based and commercial sports programs, public schools often feature sports participation prominently in secondary school in the US. Participation is generally nominal in cost, though parents often must supply necessary equipment and after-school practices may involve transportation costs. Though these programs are especially common and tend to be of higher quality in better-funded school districts that tend to have students from higher-SES families, they often offer opportunities to students from lower-SES families within those districts to obtain access to university educations through sports scholarships that would otherwise be beyond their families’ means. This is very different from the situation in Germany, where community-based and commercial sports clubs for young people are completely separate from the school system. They are often inexpensive, but inevitably involve equipment purchase and transport to some location different from the participant’s school. Supporting the idea that access to, and thus participation in, sports might be greater in the United States, Physical Activity Council data (2017) indicated that healthy physical activity levels were maintained in relevant age groups by 46–49% of the population, while WHO (2012) data indicated that 27% of German children maintained healthy levels of physical activity. Other sources indicated different levels and trends, however, and definitions of ‘healthy’ activity levels and sampling may vary considerably among organizations collecting data.

Muscle development in males as they reach adulthood also seems likely involved in the positive main effects of SES on BMI and greater E variance at higher levels of SES at the older ages in both countries. The positive main effects suggest that young males of higher SES-of-origin tended to be more likely to invest actively in athletic development. This would be consistent with greater access to opportunities to do so and greater awareness of the health and status benefits of doing so. At the same time, the greater E variance at higher levels of SES suggest that seizing these opportunities is far from uniform, and, though some co-twins may choose to participate in physical activities (either mutually supportively or competitively), with resulting gene-shared environment correlation and interaction impact on their phenotypes, other co-twins may very intentionally choose different levels of physical activity, with correspondingly different gene-non-shared environment correlation and interaction impact on their phenotypes. Either way, higher SES tends to offer more opportunities.

Recall, however, that main effects and covariance were inevitably confounded in these models because twins shared SES-of-origin. What was measured as a uniformly applicable main effect in each model was actually the coefficient of a regression through the origin (including no intercept term) of the relation between SES and BMI within their shared variance, and this shared variance was omitted from the estimated variance components (Purcell 2002). The twins’ SES-of-origin was the parents’ attained SES and its covariance with their own BMIs was passed both genetically and environmentally (through gene-environment interaction, correlation, and direct effects) to the twins. This means that intergenerationally transmitted covariance was almost entirely the covariance inevitably excluded from our models. This missing covariance likely offered the most important hints about intergenerational transmission of tendencies toward obesity so we believed it important to try to characterize it.

### Follow-up interpretative analyses

We suspect that this covariance consisted of personal characteristics that contribute to SES such as cognitive abilities, self-discipline, and working and playing well with others, which parents transmit both genetically and environmentally to their children. These also contribute to maintaining lifestyles involving diet, ample exercise, and minimal exposure to uncontrollable stress that facilitate maintaining healthy weight. This in turn can minimize expression of genetic vulnerabilities to accumulating excess weight. Such a process would at least be consistent with the observations we report here, as well as with those from the reviewed adult studies based on attained SES. Varying extents to which it is present could also contribute to differences in patterns among time and place cohorts. It is also exactly the kind of process that creates gene-environment Simpson’s paradoxes such as the contrast between Armour and Haynie’s (2007) phenotypic association between earlier age of first sexual intercourse and higher level of delinquent behavior and Harden et al.’s (2008) opposite association after controlling genetic and family influences. If within most countries the association between SES and overweight and obesity rates substantively involves, for example, relative economic and social standing as well as ability to maintain healthy lifestyle, this kind of process could also explain the Simpson’s Paradox observation that less ‘economically developed’ countries tend to have lower rates of overweight and obesity than do those more ‘economically developed’, but the reverse tends to be the case for individuals within countries.

But it does not explain the increases in obesity rates over time termed ‘epidemic’. Emerging environmental conditions would have to accentuate the general patterns of gene–environment correlation apparently affecting genetic expression observed here and in adult studies in ways that contribute to the population-level increases in overweight and obesity that have been observed over the past 40 years or more.

If so, we reasoned that we ought to be able to see analogous patterns of SES-of-origin moderating twin BMI if we controlled both twin BMI and SES for age-adjusted mid-parent BMI. This effectively removed the inter-generationally transmitted covariance that the moderation models confound with main effects. That is, any moderating effects of the resulting SES residual on the twin BMI residual should reflect whatever it is about parental SES that is independent of parental BMI yet acts on offspring BMI to accelerate it from one generation to the next. Because twins share this residual SES too, this moderating model still omits any covariance between the twins’ levels of whatever parental characteristics contributed to their own attained SES and the twins’ BMIs. Most of the relevant SES residual–twin BMI residual correlations were very small and not significant, however, so there was likely little such covariance. The model-indicated main effects should thus reflect mostly whatever direct residual-SES-of-origin effects applied uniformly to everyone. These would directly drive the increases over time in obesity levels that have been observed.

Results of fitting such models were consistent with this. The variance moderation patterns were the same as those using full SES and full BMI, but weaker, with not all reaching significance. This was consistent with presence of environmental changes that often accelerate the kind of hypothesized gene-environment interplay process described above. Similarly, modeled main effects were almost completely consistent, though weaker. All were still significant. This was consistent with presence of uniform main effects of SES on obesity levels. These would likely be increases over time in the kinds of SES-related environmental disparities involved in that hypothesized process.

The remaining moderating effects may have reflected stronger links between SES-of-origin and especially genetic vulnerabilities to weight gain in the offspring than the parental generation. If so, they would have contributed to the increases over time in obesity rates that have been observed. Such links would likely include decreasing job opportunities for people of limited educational attainment, increasingly stressful technological and economic environments that make healthy lifestyles increasingly difficult for those with lower levels of education and greater financial pressures, and increasing social and political challenges involving massive population migrations that strain public resources in ways that tend to impact those of lower SES more.

We suggest that the extent to which the main effects were weaker may have reflected environmental circumstances that foster development of overweight and obesity that affect everyone to much the same degree that have remained rather constant over the last 40 years or so. High social emphasis on educational attainment to access good job opportunities, high social and financial rewards to those who do relative to those who do not, strong correlation between house prices and school quality, ready availability and high advertising of junk foods and their relatively low cost, lower access to fresh fruits and vegetables in lower-SES neighborhoods, high proportions of sedentary jobs, and accessibility of socially and financially viable lifestyles involving little or no physical exertion, etc., are plausible examples. We also suggest that the extents to which there remained apparent main effects may have reflected increases in such circumstances over that period.

There were three exceptions to the consistency in main-effect patterns with removal of parental transmission. They were in the older male age groups in which the full-model main effects in both countries were particularly weak. In MTFS males at 17 and 24 and TwinLife Cohort 4 (ages 23–24) the directions of main effects in these groups were reversed. We suggest that this may have reflected environmental circumstances that have emerged between the parent and offspring generations that affect young men’s greater tendencies to gain muscle mass rather than adiposity in higher SES environments than in lower. Increasing popularity of endurance and extreme sports and body-building seems a likely possible contributor.

**Fig. 6:**
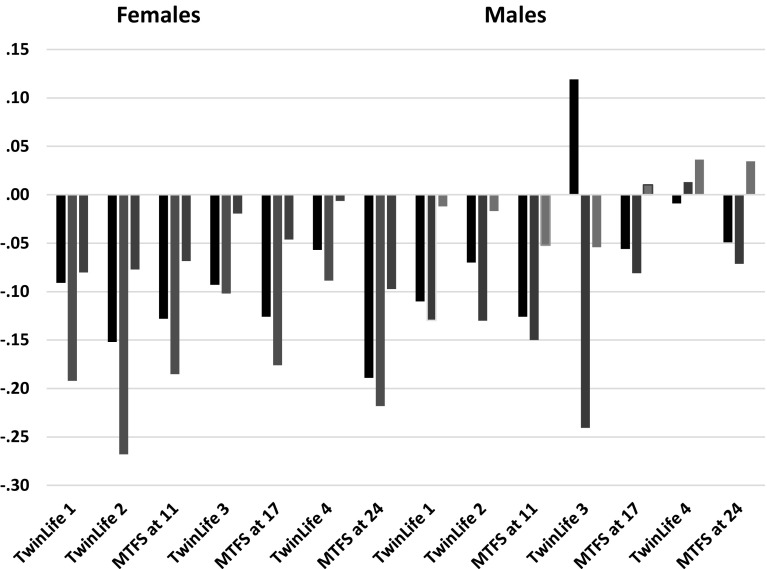
Black bars indicate the phenotypic correlations between BMI and full SES-of-origin, red bars the model-indicated main effects in their full covariances for female (blue for male), and purple bars the model-indicated main effects in the residual SES-residual BMI covariances for female (green for male) twins from TwinLife in Germany and MTFS in Minnesota. Digits for samples refer to ages

Figure 6 compares the phenotypic correlations between BMI and full SES-of-origin, the model-indicated main effects in their full covariances, and the model-indicated main effects in the residual SES-residual BMI covariances in each sex, age group, and country. We highlight this comparison rather than the moderating effects because we suspect that these main-effect indications are the more powerful drivers of the increasing rates of overweight and obesity and their associations with SES. The phenotypic correlations summarize the associations between the variables across the full range of SES as if they were constant in magnitude. This confounds direct effects of SES on BMI that apply uniformly to everyone with sometimes offsetting, sometimes enhancing moderating effects on genetic and environmental components as well as gene-environment covariance. This likely understates direct uniform main effects. The model-indicated main effects in the full models treat all the covariances as if they were only direct main effects, likely overstating direct uniform main effects. The model-indicated main effects in the parental BMI–SES-residualized covariances do the same, but the extent of covariance there was considerably reduced.

Again with the exception of the older male groups, the model-indicated main effects from the full models were more strongly negative than the phenotypic correlations, consistent with the former overstating actual direct uniform main effects and the latter understanding them. In girls, differences were especially pronounced at the younger ages, suggesting greater underlying gene-environment correlation. This would be consistent with more home-based lives in ways that matter for BMI at younger ages. The model-indicated main effects from the full models were stronger in the younger female TwinLife cohorts than the older, but stronger at age 24 in MTFS, suggesting some country-level differences. The younger boys from both countries showed similar patterns to the females, but to less striking degrees. As discussed above, the model-indicated main effects from the residualized models were the weakest of the three in all these groups. And we have also addressed the rather disparate patterns among the older male groups *Summary and conclusions*.

## Conclusions

We assessed and compared height and weight tendencies, prevalences of overweight and obesity, and extents to which SES-of-origin moderated BMI in two generally comparable and population-representative samples of twins of various young ages and their parents, one from Germany and the other from Minnesota in the US. Such direct comparisons of phenotypes and patterns of underlying genetic and environmental influences in two places and/or times are important because, while we hope for similarities that might indicate generalities in heritabilities, we should expect some differences as well. The literature on the heritability of BMI has indicated that this can take quite a range in different populations (e.g., Min et al. 2013; Silventoinen et al. 2010). Genes influence the environments people experience and how they respond to those environments, and differences between places and/or times may be even more informative about processes than similarities. Our samples were probably as suited for this purpose as is practically possible without actively cooperating on study design. They were closely matched on population-representativeness, family sampling, and measurements, and access to parental as well as twin BMI data was a clear advantage over most studies. As always, however, there were weaknesses as well. MTFS participants were assessed considerably earlier in time than TwinLife’s, and over a much longer time period, introducing potential timing-of-assessment effects within it, and cohort differences from TwinLife. We controlled within-study effects to the extents possible, but could not control the country-timing confound. In both studies, parents were slightly more educated on average than their source populations, but to similar degrees.

Our specific study purposes were to extend the literature in this area that has been based on attained SES in adults by considering developmental trends and inter-generational transmission, and comparing closely analogous observations from two countries to help identify factors contributing to the world-wide obesity epidemic and its increasing association with low SES within countries but high SES among countries. In the process, we tabulated and compared overweight and obesity rates in parents and in the various age-sex groupings of twins in the two countries according to both WHO and CDC guidelines for children.

Mean levels of adult parental BMI and prevalence rates of overweight and obesity were generally consistent with WHO and CDC adult statistics, indicating higher weights and BMIs in the US than in Germany, but neither sample was completely consistent with their reports of higher overweight and obesity levels in women than men, and TwinLife rather consistently indicated the opposite. A possible explanation for this is that muscle tissue weighs more than fat tissue, and men tend to accumulate more of it. As both samples were somewhat better educated than their source populations, an increasing worldwide tendency to pay greater attention to maintaining physical fitness in adulthood could explain our observations in both samples, as the German sample was surveyed considerably more recently. Alternatively, but not mutually exclusively, our observations could reflect a persistent country-level relative sex difference in maintenance of physical fitness in adulthood.

The most striking sex and country differences, however, lay in variance. Though adult height variance was greater in the German than the Minnesota sample, the reverse was consistently the case for weight and BMI in both twins and parents. As well, females consistently showed greater variance in weight and BMI than males, but in general males showed greater variance in height.

Second, rates of overweight and obesity in young adult twins lagged their parents’ considerably, but were higher than their younger counterparts’. The younger twins’ rates evidenced the weaknesses of BMI as a measure of overweight and obesity, as well as general developmental patterns, sex differences in those patterns surrounding pubertal timing and muscle accumulation, and country differences. They also revealed considerable differences in indicated prevalences of overweight and obesity based on WHO and CDC guidelines, which was the subject of our third research question. These differences clearly reflected the specifics of both the distributional properties of our samples and those of the samples on which the WHO and CDC based their guidelines. Both their approaches to identifying clinical cut-offs seem unhelpfully disconnected from any actual health implications of youth overweight and obesity.

Fourth and fifth, parent–offspring correlations indicated both genetic and family lifestyle transmission, as well as generation-specific effects and developmental processes. Patterns were similar in the two countries and sexes. Most of the variance in BMI could be attributed to genetic influences in both sexes and countries at all ages, but shared environmental influences were also important at the younger ages, especially in TwinLife and in girls.

Addressing our sixth and seventh questions, SES-of-origin was negatively associated with BMI in almost all age groups in both sexes in both countries, and the one exception seemed likely to indicate weaknesses in BMI as a measure of overweight and obesity rather than an exception to the general SES–BMI association. SES-of-origin moderated independent variance in youth BMI in ways very similar to the observations based on attained SES in adults, with less variance at higher levels of SES, and most of the moderation on genetic variance. Our observations were also quite, though not completely, consistent across sexes, age groups, and countries. We interpreted the similarities as suggesting that lifestyles associated with personal characteristics fostering SES attainment, passed from generation to generation, facilitate maintenance of healthy body weight as well. We offered several possible reasons for the differences that require further testing.

Finally, we explored whether our data could help to explain the increases in overweight and obesity over the past 50 years or so that have been observed. By regressing parental BMI from both SES and twin BMI and fitting the resulting SES-moderation models, we obtained both suggestions of considerable consistency in the patterns affecting parent and offspring generations and indications that these patterns had increased in strength from one generation to the next. This deserves further exploration and testing too.

## Electronic supplementary material

Below is the link to the electronic supplementary material.

Supplementary material 1 (XLSX 48 KB)
